# Physician knowledge of a rare foot condition – influence of diabetic patient population on self-described knowledge and treatment

**DOI:** 10.1186/s40842-017-0041-4

**Published:** 2017-02-08

**Authors:** Brian M. Schmidt, James S Wrobel, Crystal M. Holmes

**Affiliations:** Department of Internal Medicine, Division of Metabolism, Endocrinology, and Diabetes, University of Michigan Hospital and Health Systems, Domino’s Farms (Lobby C, Suite 1300) 24 Frank Lloyd Wright Drive Ann Arbor, Michigan, USA

## Abstract

**Background:**

Charcot neuroarthopathy (CN), a rare foot and ankle condition usually complicating diabetes mellitus, leads to deformity, poor quality of life, and increased mortality and morbidity. The prevalence of this condition in the diabetic patient population is not currently known but has been reportedly misdiagnosed in up to 95% of cases.

**Methods:**

We sought to evaluate general knowledge regarding CN in non-foot specialist clinical faculty at a large academic institution and to understand their practice habits. Our survey emphasizes the necessity of better education surrounding CN to improve outcomes in a preventative fashion. This will enable us to determine how to focus educational forums surrounding this topic in the future.

**Results:**

Seven hundred eighty-nine faculty members were sent the survey while 400 completed the survey for a response rate of 50.7%. The respondents were representative of academic rank at the institution and were comprised of endocrinologists, internal medicine physicians and family medicine physicians. We found that 67.6% of responders had a self-described poor or complete lack of knowledge of this condition. Clinicians with self-described better knowledge of CN were more likely to provide a correct initial management of CN (*p* < 0.001; *r* = 0.3639).

**Conclusions:**

In this large tertiary institution, a majority of providers among internal medicine, endocrinologists, and family medicine physicians demonstrated minimal or no knowledge of this rare, but potentially devastating diabetes complication. However, those providers who are knowledgeable of CN, performed better in the initial management of this condition. Also, respondents who treated more diabetic patients demonstrated an association with correct management. Education, and the development of better understanding amongst clinicians, is crucial to limit the devastating effects of this condition in the future.

**Electronic supplementary material:**

The online version of this article (doi:10.1186/s40842-017-0041-4) contains supplementary material, which is available to authorized users.

## Background

Charcot neuroarthopathy (CN), is a rare foot and ankle condition [1] that can lead to both structural and functional abnormalities resulting in ulceration and amputation. CN has an overall incidence of 0.1% - 0.9% and it is more commonly thought to be seen, relatively, in the diabetic population [2–5]. However, since there is not an unambiguous definition or diagnosis for CN, true incidence and prevalence data is not currently known [1]. Past studies have demonstrated that referring physicians can misdiagnosis CN up to 95% of the time when referring to a foot specialist [6–8]. When an accurate diagnosis is made and treated within 8 weeks of onset, there is a five-fold reduction in complications from 67% to 14% [6]. It has been estimated that the average diagnostic delay is up to 29 weeks [9]. More astute recognition of CN is critical given both the rarity of this condition and rapid escalation in foot collapse excess morbidity experienced by these patients following misdiagnosis or delayed diagnosis [2–4, 6].

There is a lack of literature about general knowledge of CN amongst physicians. A search in PubMed/NCBI, Google Scholar, and Cochrane Databases for Charcot foot education in physician reveals no studies on the specific subject. Therefore, the objective of this study was to evaluate general CN knowledge in the diagnosis and initial management among non-foot specialist clinicians in a large academic based-practice.

## Methods

A fourteen item questionnaire was developed (Additional file 1) with an online tool (Qualtrics, www.qualtrics.com). Questions were formulated to better understand clinical knowledge of CN in relation to differential diagnosis, imaging, diagnosis and classification, clinical tests, and management. There were also questions regarding experience and training. The questionnaire was designed to only take 3 min or less to complete and was sent to all internal medicine clinical faculty members and family medicine at the University Health System. All responses were anonymous and respondents could only take the survey once. The only identifying condition of the survey response was the IP address of the computer or mobile device where it was accessed. Qualtrics reports information about the e-mail itself, such as: if it was opened, when the survey was started, when the survey was completed, the time it took the respondent to complete the survey, and if the survey was completed or only partially completed. The local institutional review board approved the study.

### Analyses

We excluded all partially completed surveys. Statistical analysis consisted evaluation of trends between and among groups. The Pearson correlation coefficient was used to quantify correlation, if any, between queried relationships. All statistical analysis was completed using SPSS statistical software, version 22 (SPSS Inc., Chicago, IL). Statistical significance was set at the 5% level (*p* ≤ 0.05).

## Results

The survey was sent to 789 clinical faculty members. Qualtrics reported a delivery of 100% of the survey to the desired e-mail locations. 448 surveys were started and 400 surveys were completed (89.3%). Non-responders did not attempt survey. The overall response rate was 50.7%. Forty-eight surveys were only partially completed and each of them was less than 50% complete. The data from these incomplete surveys were excluded from analysis.

For medical specialty, there were 56.9% internal medicine, 17.2% family medicine, 12.1% endocrinologists, 5.9% rheumatologists, and the remaining 6.9% responded as other, including pulmonary, palliative medicine, oncology, plastic surgery, and infectious disease. Academic rank of those completing the survey, included: 3.2% fellows, 9.6% clinical lecturer, 10.3% clinical instructor, 44.1% assistant professor, 16.2% associate professor, and 16.7% were at the professor level. There were no disparate responses amongst rank when compared to medical school data.

When we asked the individuals responding to the survey to describe their knowledge of CN only 6.9% reported having excellent knowledge while 5.0% reported as good, and 20.5% reported fair, while the remaining 67.6% reported having a poor or no knowledge of CN. Academic rank demonstrated a significant association (*p* < 0.05; *p* = 0.0422) with self-described CN knowledge although correlation was weak (*r* = 0.1070). Specialty did not demonstrate a significant association with CN knowledge (*p* > 0.05; *r* = 0.1228).

We inquired about diabetes patient panel size. We received 375 responses. 49.1% (*n* = 184) selected 0-25%, 36.5% (*n* = 137) selected 25-50%, 8.0% (*n* = 30) choose 50-75%, and 3.7% (*n* = 14) selected 75-100% of patients having diabetes mellitus. There was no significant association between rank and/or specialty reported with percentage of diabetic patients seen.

Of the 44 who chose greater than 51% (majority) treat persons with diabetes in their clinics, the vast majority of these physicians (*n* = 37, 84.1%) choose offloading as treatment for a patient with CN while the remaining 7 choose referral (15.9%). This is in contrast to physicians who treat less than 50% of people with diabetes in their clinic. Of the 317 who answered in this way, 156 (49.2%) chose offloading, 143 (45.1%) choose referral, and 18 (5.7%) chose no treatment. However, significant difference between these groups was not realized (*p* > 0.05).

We found a significant inverse relationships between percent patients with diabetes patients seen and self-reported knowledge of CN (*p* < 0.001, *r* = −0.3149) and understanding that peripheral neuropathy is required for a CN event (*p* < 0.001, *r* = −0.1962). Providers seeing more diabetes patients were significantly more likely to select offloading as treatment modality of choice (*p* = 0.0014, *r* = −0.2307). The association was independent of self-assessed CN knowledge (*p* < 0.001; r =0.3639).

## Discussion

Charcot neuroarthopathy is often misdiagnosed [6–8] which can lead to severe sequelae including limited mobility, ulceration, infection, amputation, decreased quality of life, and increased mortality [10, 11]. Previous CN intervention has been done in later stages with costs ranging from $49,251 to $56,712 for major lower extremity amputation versus reconstructive limb salvage, respectively [12]. Despite advances in limb salvage including surgery we still see devastating effects. This may be the result of lack of recognition of the condition entirely.

We feel that a better target is physician education and prevention of the debilitating limb threatening deformities. We sought to evaluate CN knowledge of non-foot specialist providers at a large academic institution to identify gaps in knowledge in preparation for future clinician related education programs. The sample included diversity in physician rank and practice including: internal medicine, family medicine, rheumatologists, endocrinologists, among others.

Not surprisingly, we found that most survey respondents, approximately 67.6%, had a poor or no general self-described knowledge of CN. Frequently, early changes of CN are not recognized by the patient as they are neuropathic. Upon initial evaluation as there are no gross foot deformities by exam or x-ray, and subtle joint temperature increases (~9 °F) [13] cannot be appreciated by many clinicians. When referrals delays are factored in, it is not surprising that many CN patients experience excess morbidity and mortality [14].

Self-described knowledge of CN was associated with correct initial management (*p* < 0.001; *r* = 0.3639) of offloading. We thought that physicians who saw more diabetic patients would also correctly treat a patient with CN, as it is a complication in this population. This was largely confirmed by analyzing groups who see a majority of diabetic patients in their clinic versus those who do not although our findings did not reach statistical significance. Nevertheless, we attribute this to weak powering in the group of physicians who see majority diabetic patients in their clinics. Subsequently, we did demonstrate that there is a significant trend between percent people with diabetes and correctly managing a patient with CN.

## Conclusion

CN is a disease which afflicts patients with small fiber peripheral neuropathy. If not appropriately diagnosed and treated quickly, the consequences can be significant and lead to increased patient morbidity and mortality. In our survey, we identified most respondents from a large academic institution do not have a self-described general knowledge of CN and this may further lead to the practitioners (in-) ability to correctly diagnose CN. Furthermore, we demonstrate that the better one’s knowledge is of CN, the more apt that physician is to correctly treat the condition initially with offloading. Also, larger diabetes panel sizes demonstrated an association with managing this subset of patients correctly. Our study highlights the importance of education and demonstrates the need for more educational and awareness programs for referring providers about CN as well as more efficient referral processes.

## Additional file

Additional file 1: Physician Survey about Knowledge of a Rare Foot Condition. (DOCX 24 kb)

## Abbreviation

CN: Charcot Neuroarthropathy
