# Supplementary material for: Physician knowledge of a rare foot condition – influence of diabetic patient population on self-described knowledge and treatment
Source: Clin Diabetes Endocrinol. 2017 Feb 8;3:2. doi: 10.1186/s40842-017-0041-4 (PMC5471700; doi:10.1186/s40842-017-0041-4)
Supplement: Additional file 1: — Physician Survey about Knowledge of a Rare Foot Condition. (DOCX 24 kb) [file 40842_2017_41_MOESM1_ESM.docx]

Physician Survey about Knowledge of a Rare Foot Condition

Return to:

Brian M Schmidt, DPM

Clinical Instructor

Department of Internal Medicine

Division of Metabolism, Endocrinology, and Diabetes

24 Frank Lloyd Wright Drive

Lobby C – 1300

Ann Arbor, MI 48106

**Please answer all questions to the best of your ability**

Q1. Which best describes your current position at the University of Michigan? (Choose ONE)

- Student
- Resident
- Fellow
- Faculty
  - Professor
  - Associate Professor
  - Assistant Professor
  - Clinical Instructor
  - Clinical Lecturer

Q2. Which choice best describes your medical specialty? (Choose ONE)

- Internal Medicine
- Family Medicine
- Endocrinology
- Podiatry
- Orthopedics
- General Surgery
- Vascular Surgery
- Rheumatology
- Other

Q2a If you selected “Other” above, please list your specialty. If not, please proceed to Q2.

- Other: ________________________________

Q3. If an erythematous, edematous, and warm foot presents to your clinic, what are your top three differential diagnoses? (Choose THREE)

- Gout
- Charcot Neuroarthropathy
- Cellulitis
- Osteomyelitis
- Charcot Marie Tooth Disease
- Fracture
- Deep Vein Thrombosis
- Sprain
- Septic Arthritis

Q4. What approximate percentage of your clinic patient population has diabetes mellitus, Types 1 and 2? (Choose ONE)

- 0% – 25%
- 26% - 50%
- 51% - 75%
- 75% - 100%

Q5. If a patient with diabetic neuropathy presents with an erythematous, edematous, and warm foot, what are your top three differential diagnoses? (Choose THREE)

- Gout
- Charcot Neuroarthropathy
- Cellulitis
- Osteomyelitis
- Charcot Marie Tooth Disease
- Fracture
- Deep Vein Thrombosis
- Sprain
- Septic Arthritis

Q6. How would you describe your knowledge of Charcot Neuroarthropathy? (Choose ONE)

- Excellent
- Good
- Fair
- Poor
- No practical knowledge
- Other

Q7. Do all patients with Charcot Neuroarthropathy have peripheral neuropathy?

- Yes
- No
- I don’t know

Q8. Which physical examination tools are most sensitive to identify signs of small fiber peripheral neuropathy? (Choose TWO)

- Semmes Weinstein monofilament testing
- Vibratory sensation
- Deep tendon reflexes
- Sharp/dull sensation
- Proprioception
- Light touch
- Two point discrimination
- Temperature discrimination

Q9 Do you know the stages of Charcot Neuroarthropathy?

- Yes
- No

Q10. If a patient is presenting with an erythematous, edematous, and warm foot, and your differential includes Charcot neuroarthropathy, what one imaging study would you order? (Choose ONE)

- Radiograph bearing weight
- Radiograph not bearing weight
- MR
- CT scan
- DEXA scan
- Tomography
- PET scan
- Bone scan

Q11. If you suspected Charcot Neuroarthropathy, who you would refer to? (Choose ONE)

- Foot and Ankle Orthopedist
- Orthopedist
- Podiatrist
- Foot Specialist
- General Practitioner
- Emergency Room
- Vascular Surgery
- No referral

Q12. If you suspected Charcot Neuroarthropathy, what would your initial treatment regimen consist of? (Choose ONE)

- Regular Shoe Gear
- Antibiotics
- Offloading
- Rest, ice, compression, elevation (RICE)
- No treatment
- Referral

Q12a. What would be the offloading device of choice for a patient with Charcot Neuroarthropathy?

- Wheelchair
- Cast
- Walking Boot
- Crutches

Thank you for your participation. Results will be presented at a later date.
